# The need for nutritional assessment and interventions based on the prognostic nutritional index for patients with femoral fractures: a retrospective study

**DOI:** 10.1186/s13741-021-00232-1

**Published:** 2021-12-20

**Authors:** Miao He, Qinghong Fan, Yuhang Zhu, Dexing Liu, Xingxing Liu, Shan Xu, Jiachen Peng, Zhaoqiong Zhu

**Affiliations:** 1 Medical College of Soochow University, Suzhou, Jiangsu China; 2 Department of Anesthesiology, Affiliated Hospital of Zunyi Medical University, 149 Da Lian Road, Hui Chuan District, Zunyi, 563003 China; 3 Department of Anesthesiology, Clinical Medical College and Affiliated Hospital of Chengdu University, Chengdu, Sichuan China; 4 Department of Orthopedics, The Second Affiliated Hospital of Zunyi Medical University, Zunyi, Guizhou China; 5 Department of Orthopedics, Affiliated Hospital of Zunyi Medical University, Zunyi, Guizhou China

**Keywords:** Prognostic nutritional index, Femoral fracture, Adverse perioperative outcomes, Independent factors, Nutrition

## Abstract

**Background:**

The incidence of adverse perioperative outcomes in surgery for femoral fractures is high and associated with malnutrition. Here, we identified independent factors and assessed the predictive value of the prognostic nutritional index (PNI) for perioperative adverse outcomes in patients with femoral fractures.

**Methods:**

This retrospective study included 343 patients who underwent surgery for a single femur fracture. Demographic characteristics, surgery and anaesthesia records and blood test results at admission, 1 day postoperatively and before discharge were evaluated using logistic regression analysis. The discriminatory ability of the independent factors was assessed using the receiver operating characteristic curve analysis, and DeLong’s test was used to compare the area under the curve (AUC).

**Results:**

Overall, 159 patients (46.4%) experienced adverse perioperative outcomes. Amongst these, 123 (35.9%) had lower limb vein thrombus, 68 (19.8%) had hospital-acquired pneumonia, 6 (1.7%) were transferred to the postoperative intensive care unit, 4 (1.2%) had pulmonary embolism, 3 (0.9%) died during hospitalisation and 9 (2.6%) had other adverse outcomes, including incision disunion, renal and liver function impairment, acute heart failure, acute cerebral infarction and stress gastroenteritis. The PNI at admission, age, postoperative hospital stay, time to admission, hypertension, combined injures and surgery type were independent factors for adverse perioperative outcomes. Based on the AUC (PNI at admission: 0.772 [0.723–0.821], *P* < 0.001; age: 0.678 [0.622–0.734], *P* < 0.001; postoperative hospital stay: 0.608 [0.548–0.668], *P* = 0.001; time to admission: 0.585 [0.525–0.646], *P* = 0.006), the PNI at admission had optimal discrimination ability, indicating its superiority over other independent factors (age vs. PNI at admission, *P* = 0.002; postoperative hospital stay vs. PNI at admission, *P* < 0.001; time to admission vs. PNI at admission, *P* < 0.001).

**Conclusions:**

Patients with femoral fractures require a nutritional assessment and appropriate nutritional intervention at admission, and that the PNI value at admission may be a good nutritional assessment indicator.

## Background

The number of individuals sustaining fractures is increasing globally, particularly amongst the elderly population (Melton 3rd., 1993; Hughes et al. 2006). Clinically, femoral fractures are one of the most encountered fracture types and are associated with higher rates of complications, profound reduction in quality of life and increase in morbidity, mortality and economic costs (Hughes et al. 2006; Haentjens et al. 2003; Amarilla-Donoso et al. 2020). Femoral fractures are generally classified according to the site of fracture as proximal, shaft or distal femoral fractures. The incidence of proximal femoral fractures, classified as hip fractures, is the highest and is likely to continue to increase in the future owing to the rapidly ageing population and associated occurrence of osteoporosis (Giancola et al. 2016; Lutnick et al. 2020; Cummings and Melton 2002). Femoral shaft fractures, which are predominantly noted in young people with healthy bones (Cummings and Melton 2002), are primarily caused by road traffic accidents (being crushed or run over) or falling from a great height (Gosling and Krettek 2019). Distal femoral fractures are rare injuries, accounting for approximately 2% of all femoral injuries (Young and Stans 2018), and often develop due to vehicular trauma or sports activities with varus or valgus impact at the knee (Young and Stans 2018).

Surgery is usually the best treatment, and it is often performed for patients with femoral fractures. However, the incidence of adverse perioperative outcomes is quite high, including lower limb vein thrombus or pulmonary embolism, pneumonia, incision disunion or infection, acute exacerbation of underlying chronic diseases, transfer to the intensive care unit and even death. Age, trauma, stress, surgery, anaemia, bleeding, infection, pain, activity limitation and a bedridden state are commonly considered to be the causes for the aforementioned outcomes. Such patients may be at risk for protein catabolism and malnutrition. Furthermore, nutrition has a major influence on fracture healing, and fracture healing impairment has been observed amongst malnourished and undernourished individuals (Hughes et al. 2006; Meesters et al. 2018; Invernizzi et al. 2019). Protein-depleted patients with a hip fracture have shown higher complication rates and longer hospitalisation periods (Meesters et al. 2018). Notably, Hughes et al. have shown that nutritional improvement leads to increased muscle mass in the leg and greater bone mineral density in the fractured callus in protein-malnourished rats with femoral fractures (Hughes et al. 2006). Nevertheless, only a few studies have investigated the impact of nutrition on adverse outcomes in patients with femoral fractures, specifically during the perioperative stage.

The prognostic nutritional index (PNI), initially proposed by Buzby et al. (1980), is a comprehensive index for evaluating the nutritional status of patients undergoing surgery (Caputo et al. 2020; Ren et al. 2017). The PNI can be used to assess the nutritional and immunological status of patients undergoing surgery, and can be estimated according to the following formula: 10 × serum albumin (ALB, g/dl) + 0.005 total lymphocyte count (LYM, per mm^3^). Currently, a low PNI, as a proxy for subpar perioperative nutritional status, is reportedly a significant predictor of poor postoperative outcomes and increased mortality in various malignancies (Cadwell et al. 2020; Li and Chen 2019). However, studies on PNI focusing on the perioperative adverse outcomes of patients undergoing surgery for femoral fractures are almost non-existent. Therefore, in this retrospective study, we aimed to determine the independent factors for perioperative adverse outcomes and evaluate the predictive value of the PNI in patients with femoral fractures.

## Methods

### Data source

The data for this retrospective observational study were extracted from the Hospital Information System (TianJian Technology Co., Ltd., Beijing, China) and Anaesthesia Information Management System (Medical System Technology Co., Ltd., Suzhou, Jiangsu, China). The Hospital Information System and the Anaesthesia Information Management System, which maintain a complete record of healthcare services, are electronic medical record management systems for hospitals in China.

### Patients

**Fig. 1:**
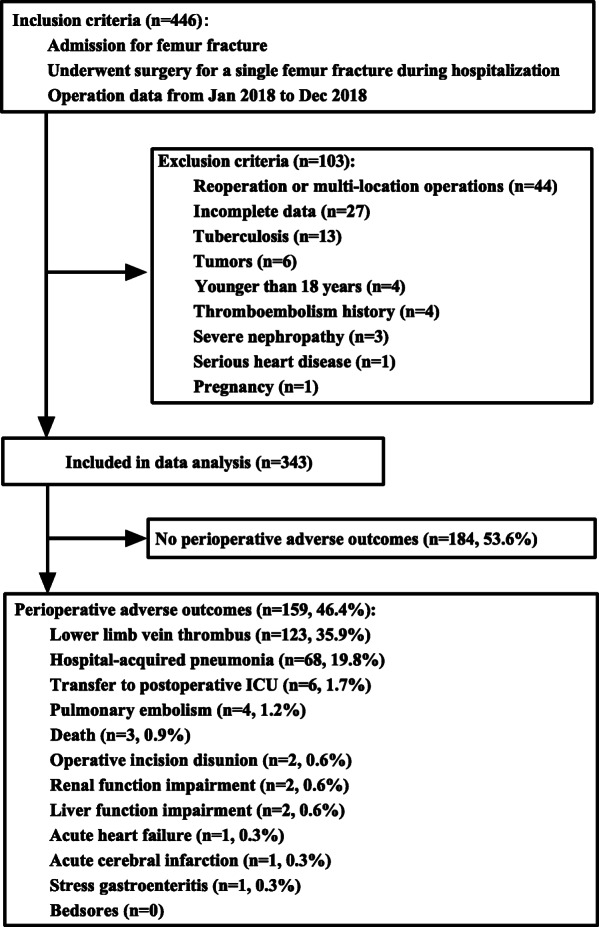
Flow chart of patient inclusion. ICU, intensive care unit

A retrospective review was performed using data from a database of 446 patients who underwent surgery for a single femur fracture during hospitalisation between January 2018 and December 2018 at the Affiliated Hospital of Zunyi Medical University. These patients did not receive nutritional counselling during hospitalisation. The main nutritional interventions, such as infusion of ALB, amino acid and fat emulsion, were used as conventional therapies only when the ALB concentration was < 30 g/l. The case definition of femur fracture was based on specific diagnosis codes from the International Classification of Diseases, Tenth Revision (ICD-10, S72). These codes were listed as the primary diagnosis on the electronic inpatient healthcare claim submitted to the Hospital Information System. The exclusion criteria were as follows: (i) reoperation or surgeries at multiple sites (*n* = 44); (ii) incomplete data (*n* = 27); (iii) systemic wasting diseases (tuberculosis, tumours and hyperthyroidism; *n* = 19); (iv) age <18 years (*n* = 4); (v) history of thromboembolism (*n* = 4); (vi) chronic renal failure, chronic hepatic dysfunction and serious heart disease (*n* = 4); and (vii) pregnancy (*n* = 1; Fig. 1). Finally, a total of 343 patients were identified after applying all exclusion criteria; amongst these, 257 (75.0%) had a proximal fracture, 79 (23.0%) had a shaft fracture and 7 (2.0%) had a distal fracture.

### Ethics approval

This retrospective study was conducted in accordance with the principles outlined in the Declaration of Helsinki and was approved by the Research Ethics Committee of Affiliated Hospital of Zunyi Medical University (reference number: KLL-2020-022). Informed consent was waived by the Research Ethics Committee of Affiliated Hospital of Zunyi Medical University due to the anonymous nature of the data.

### Perioperative adverse outcomes

Perioperative complications, such as lower limb vein thrombus (ICD-10, I80.301), pulmonary embolism (ICD-10, I26), hospital-acquired pneumonia (ICD-10, J12-18), incision disunion (ICD-10, T81.406 or T81.009), bedsores (ICD-10, L89) and transfer to the intensive care unit and death (ICD-10, R99), were defined as adverse perioperative outcomes. The observation period was from admission to discharge.

### Variables

The following various potential influencing factors were investigated: (i) demographic characteristics, including sex, age, weight, chronic diseases, combined injuries, aetiology of fracture, fracture site, postoperative hospital stay and time to admission (time to admission was graded as 1 (within 24 h), 2 (2–3 days), 3 (4–7 days), 4 (8–21 days) or 5 (> 22 days)); (ii) surgery and anaesthesia records, including the American Society of Anesthesiologists (ASA) grade, surgeons (eight chief surgeons with at least 20 years of surgical experience (surgeons A, B, C, D, E, F, G and H) participated in this study), surgery type, anaesthesia methods, postoperative analgesic methods, duration of anaesthesia and surgery, ratio of perioperative blood transfusion, intraoperative blood loss and intraoperative crystal and colloidal liquid infusion volumes; and (iii) laboratory results at admission, 1 day postoperatively and before discharge (“before discharge” was defined as the results of the latest blood test, usually within 48 h before discharge), including ALB, prealbumin (PAb), globulin (GLB) and Hb concentrations, LYM and neutrophil (NEUT) counts, neutrophil to lymphocyte ratio (NLR) and PNI (Table 1).

**Table 1:** Comparison between patients with and without adverse outcomes

Variables	No adverse outcomes (*n* = 184)	Adverse outcomes (*n* = 159)	*P* values
Male/female, *n* (%)	88 (47.8)/96 (52.2)	70 (44.0)/89 (56.0)	0.481
Left/right femur, *n* (%)	94 (51.1)/90 (48.9)	78 (49.1)/81 (50.9)	0.708
Age, years, mean (range)	60 (43.25–71)	70 (60–81)	**< 0.001**
Weight, kg, mean (range)	55 (50–62)	55 (50–60)	0.259
Hypertension, *n* (%)	45 (24.5)	74 (46.5)	**< 0.001**
Diabetes, *n* (%)	12 (6.5)	18 (11.3)	0.117
Combined injuries, *n* (%)	112 (43.1)	47 (56.6)	**0.031**
Aetiology, *n* (%)			
Sprain or tumble	142 (77.2)	123 (77.4)	0.968
RTA, falls or assaults	42 (22.8)	36 (22.6)	
Fracture site, *n* (%)			
Proximal femoral fracture	140 (76.1)	117 (73.6)	0.493
Femoral shaft fracture	39 (21.2)	40 (25.2)	
Distal femoral fracture	5 (2.7)	2 (1.3)	
Time to admission, *n* (%)			
1: Within 24 h	127 (69.0)	81 (50.9)	**0.002**
2: 2–3 days	17 (9.2)	24 (15.1)	
3: 4–7 days	14 (7.6)	19 (11.9)	
4: 8–21 days	12 (6.5)	26 (16.4)	
5: ≥ 22 days	14 (7.6)	9 (5.7)	
ASA, *n* (%)			
I	4 (2.2)	2 (1.3)	**< 0.001**
II	157 (85.3)	106 (66.7)	
III	23 (12.5)	50 (31.4)	
IV	0 (0)	1 (0.6)	
Surgeons, *n* (%)			
Surgeon A	28 (15.2)	22 (13.8)	0.161
Surgeon B	38 (20.7)	21 (13.2)	
Surgeon C	22 (12.0)	18 (11.3)	
Surgeon D	27 (14.7)	22 (13.8)	
Surgeon E	31 (16.8)	32 (20.1)	
Surgeon F	13 (7.1)	17 (10.7)	
Surgeon G	18 (9.8)	11 (6.9)	
Surgeon H	7 (3.8)	16 (10.1)	
Operation types, *n* (%)			
Hemi/total-hip hip replacement	73 (39.7)	43 (27.0)	**0.014**
Internal fixation	111 (60.3)	116 (73.0)	
Anaesthesia methods, *n* (%)			
General anaesthesia	47 (25.5)	35 (22.0)	0.445
Non-general anaesthesia	137 (74.5)	124 (78.0)	
Postoperative analgesic methods, *n* (%)			
No postoperative analgesia	9 (4.9)	4 (2.5)	0.315
PCIA	123 (66.8)	101 (63.5)	
PCEA	52 (28.3)	54 (34.0)	
Duration of anaesthesia, min, mean (range)	180 (140–225)	180 (149–230)	0.323
Duration of surgery, min, mean (range)	111 (80–153.5)	110 (80–155)	0.914
Blood transfusion, *n* (%)	61 (33.2)	87 (54.7)	**< 0.001**
Intra-blood loss, × 10^2^ ml, mean (range)	2 (1–3)	2 (1–3)	**0.052**
Intra-crystal liquids, × 10^2^ ml, mean (range)	8.5 (6–11)	7.5 (6–11)	0.662
Intra-colloidal liquids, × 10^2^ ml, mean (range)	5 (5–7.5)	5 (5–10)	0.983
Postoperative hospital stay, days, mean (range)	6 (4–7)	7 (5–9)	**< 0.001**
Blood test at admission			
ALB, g/l, mean ± SD	38 ± 3.91	34.48 ± 4.34	**< 0.001**
PAb, mg/l, mean (range)	191 (154–229.5)	158 (126–194)	**< 0.001**
Hb, g/l, mean ± SD	120.02 ± 18.96	108.24 ± 20.99	**< 0.001**
GLB, g/l, mean (range)	27.15 (23.35–29.95)	27.70 (24.90–30.80)	0.110
LYM count, × 10^9^/l, mean (range)	1.30 (0.98–1.68)	1.12 (0.87–1.50)	**0.004**
NEUT count, × 10^9^/l, mean (range)	5.52 (4.31–7.16)	4.97 (3.92–6.43)	**0.035**
NLR, mean (range)	4.21 (2.96–6.09)	4.71 (3.27–7.20)	0.150
PNI, mean ± SD	44.54 ± 5.05	39.30 ± 4.99	**< 0.001**
Blood test at 1 day postoperatively			
ALB, g/l, mean (range)	29.2 (27.2–32.58)	27.4 (24.6–29.3)	**< 0.001**
PAb, mg/l, mean (range)	104 (75–147)	86 (66–114)	**< 0.001**
Hb, g/l, mean (range)	91 (78–99.75)	83 (72–93)	**< 0.001**
GLB, g/l, mean (range)	22.70 (19.20–25.28)	21.40 (19.10–24.00)	**0.045**
LYM count, × 10^9^/l, mean (range)	0.84 (0.67–1.14)	0.73 (0.53–0.97)	**0.001**
NEUT count, × 10^9^/L, mean (range)	7.81 (5.92–10.01)	8.40 (6.60–11.41)	**0.026**
NLR, mean (range)	9.06 (5.86–13.49)	10.20 (8.07–14.86)	**0.001**
PNI, mean (range)	34.15 (31.55–37.61)	31.70 (29.05–34.20)	**< 0.001**
Blood test before discharge			
ALB, g/l, mean ± SD	32.75 ± 3.44	32.32 ± 4.14	0.293
PAb, mg/l, mean (range)	112.5 (86.25–160)	104 (80–141)	**0.028**
Hb, g/l, mean (range)	93 (83–102.75)	91 (82–100)	0.129
GLB, g/l, mean (range)	24.50 (21.73–27.30)	24.20 (22.10–27.90)	0.614
LYM count, × 10^9^/L, mean (range)	1.04 (0.81–1.41)	1.04 (0.71–1.33)	0.140
NEUT count, × 10^9^/L, mean (range)	5.76 (4.36–7.30)	5.31 (4.17–7.21)	0.431
NLR, mean (range)	5.29 (3.72–8.08)	5.56 (3.78–8.29)	0.440
PNI, mean (range)	37.83 (34.75–41.05)	37.05 (33.85–41.60)	0.152

Bold text represents confounding factors with *P* < 0.10

*ALB* albumin, *ASA* American Society of Anesthesiologists, *GLB* globulin, *Hb* haemoglobin, *Intra-* intraoperative, *LYM* lymphocyte, *NEUT* neutrophil, *NLR* neutrophil to lymphocyte ratio, *PAb* Prealbumin, *PCEA* patient-controlled epidural analgesia, *PCIA* patient-controlled intravenous analgesia, *PNI* prognostic nutritional index, *RTA* road traffic accidents

### Statistical analysis

Continuous data are presented as mean with standard deviation or median (interquartile range) according to statistical distribution (assumption of normality was assessed using the Kolmogorov-Smirnov test). Categorical parameters are presented as frequencies and associated percentages. The Student’s *t* test was used to analyse normally distributed continuous variables, whereas the Mann-Whitney *U* test was utilised to examine non-normally distributed continuous variables and ordinal variables (ASA grade, surgical grades and time to admission). The chi-square or Fisher’s exact test was employed to analyse categorical variables. In these analyses, variables with unadjusted *p* < 0.10 were identified as confounding factors and were included in multivariate regression analyses to determine independent predictors of adverse perioperative outcomes. The results are expressed as OR and 95% CI. The discriminatory ability of the independent predictors was assessed using the receiver operating characteristic (ROC) curve analysis. Optimal cut-off values were obtained using the Youden index, and DeLong’s test was used to compare the area under the curve (AUC) with MedCalc statistical software version 19.3.1 (MedCalc Software Ltd., Ostend, Belgium). A *p* value of < 0.05 was considered statistically significant. All tests were two-sided. All statistical analyses were conducted using Statistical Package for Social Sciences version 17.0 (SPSS Statistics for Windows, Chicago, USA).

## Results

### Patients

A total of 159 (46.4%) patients who underwent surgery for femoral fractures experienced adverse perioperative outcomes. Amongst these, 123 (35.9%) had lower limb vein thrombus, 68 (19.8%) had hospital-acquired pneumonia, 6 (1.7%) were transferred to the postoperative intensive care unit, 4 (1.2%) had pulmonary embolism, 3 (0.9%) died during hospitalisation, 2 (0.6%) had incision disunion and 7 (2.0%) had other adverse outcomes, including renal and liver function impairment, acute heart failure, acute cerebral infarction and stress gastroenteritis (Table 1).

### Confounding and independent factors

The following factors were associated with adverse outcomes: age; hypertension; combined injuries; time to admission; ASA classification; surgery type; ratio of perioperative blood transfusion; intraoperative blood loss; postoperative hospital stay; admission values of ALB, PAb, Hb, LYM count, NEUT count and PNI; 1-day postoperative values of ALB, PAb, Hb, GLB, LYM count, NEUT count, NLR and PNI; and PAb value before discharge (all *P* values < 0.10; Table 1). All the aforementioned confounding factors, except for ALB concentrations (which showed collinearity with PNI), were included in the multivariate regression analyses to determine the independent factors associated with adverse perioperative outcomes. The PNI at admission (odds ratio [OR]: 0.850, 95% confidence interval [CI]: 0.776–0.931, *P* < 0.001), age (OR: 1.041, 95% CI: 1.016–1.066, *P* = 0.001), postoperative hospital stay (OR: 1.132, 95% CI: 1.016–1.263, *P* = 0.025), time to admission (OR: 1.343, 95% CI: 1.056–1.708, *P* = 0.016), hypertension (OR: 2.091, 95% CI: 1.116–3.916, *P* = 0.021), combined injures (OR: 2.836, 95% CI: 1.340–6.003, *P* = 0.006) and surgery type (OR: 4.625, 95% CI: 2.283–9.367, *P* < 0.001) were identified as independent factors for perioperative adverse outcomes (Table 2).

**Table 2:** Multivariate regression analyses of confounding factors

Confounding factors	OR (95% CI)	*P* values
PNI at admission (per 1)	0.850 (0.776, 0.931)	**< 0.001**
Age (per 1 year)	1.041 (1.016, 1.066)	**0.001**
Postoperative hospital stay (per 1 day)	1.132 (1.016, 1.263)	**0.025**
Time to admission (per 1)	1.343 (1.056, 1.708)	**0.016**
Hypertension (ref: no)	2.091 (1.116, 3.916)	**0.021**
Combined injuries (ref: no)	2.836 (1.340, 6.003)	**0.006**
Operation types (ref: hip replacement)	4.625 (2.283, 9.367)	**< 0.001**
ASA (per I)	1.411 (0.718, 2.773)	0.317
Blood transfusion (ref: no)	1.040 (0.500, 2.163)	0.916
Intraoperative blood loss (per 1 × 10^2^ ml)	1.042 (0.878, 1.238)	0.638
PAb at admission (per 1 mg/l)	1.000 (0.993, 1.008)	0.950
Hb at admission (per 1 g/l)	1.015 (0.990, 1.040)	0.244
LYM count at admission (per 1 × 10^9^/l)	1.520 (0.677, 3.411)	0.310
NETU count at admission (per 1 × 10^9^/l)	1.015 (0.892, 1.155)	0.822
PAb at 1 day postoperatively (per 1 mg/l)	1.009 (0.995, 1.023)	0.191
Hb at 1 day postoperatively (per 1 g/l)	0.989 (0.959, 1.019)	0.460
GLB at 1 day postoperatively (per 1 g/l)	0.990 (0.915, 1.071)	0.804
LYM count at 1 day postoperatively (per 1 × 10^9^/l)	1.016 (0.249, 4.143)	0.982
NETU count at 1 day postoperatively (per 1 × 10^9^/l)	1.055(0.936, 1.189)	0.377
NLR at 1 day postoperatively (per 1)	0.991 (0.922, 1.064)	0.797
PNI at 1 day postoperatively (per 1)	0.925 (0.822, 1.040)	0.193
PAb before discharge (per 1 mg/l)	0.997 (0.987, 1.008)	0.630

The ALB concentration was not included in the model and showed significant collinearity with PNI. Bold fonts represent independent factors with *P* < 0.05

*ALB* albumin, *ASA* American Society of Anesthesiologists, *GLB* globulin, *Hb* haemoglobin, *LYM* lymphocyte, *NEUT* neutrophil, *NLR* neutrophil to lymphocyte ratio, *PAb* Prealbumin, *PNI* prognostic nutritional index, *OR* odds ratio

### AUC and optimal cut-off values of the independent factors and ALB

The discriminatory ability of the independent factors (PNI at admission, age, postoperative hospital stay and time to admission) were assessed using the ROC curve analysis. Based on the AUC (PNI at admission: 0.772, 95% CI: 0.723–0.821, *P* < 0.001; age: 0.678, 95% CI: 0.622–0.734, *P* < 0.001; postoperative hospital stay: 0.608, 95% CI: 0.548–0.668, *P* = 0.001; time to admission: 0.585, 95% CI: 0.525–0.646, *P* = 0.006), the PNI at admission had the most optimal discrimination ability and was superior to other independent factors (age vs. PNI at admission, *P* = 0.002; postoperative hospital stay vs. PNI at admission, *P* < 0.001; time to admission vs. PNI at admission, *P* < 0.001). As the ALB concentration is a primary measure included in the PNI, there might be a relationship between the ALB and adverse outcomes. However, the PNI at admission was a better predictor (*P* = 0.038) than the ALB concentration at admission (0.736, 95% CI: 0.683–0.790, *P* < 0.001). The optimal cut-off values of PNI at admission, age, postoperative hospital stay, time to admission and ALB concentration at admission were 42.425, 55.5 years, 6.5 days, 2 days and 36.35 g/l, respectively (Table 3).

**Table 3:** Comparison of the AUC for independent factors and ALB

	AUC (95% CI)	*P*	Youden_max_	Threshold	P*
PNI at admission	0.772 (0.723, 0.821)	**< 0.001**	0.425	42.425	
Age	0.678 (0.622, 0.734)	**< 0.001**	0.250	55.5 years	**0.002**
Postoperative hospital stay	0.608 (0.548, 0.668)	**0.001**	0.196	6.5 days	**< 0.001**
Time to admission	0.585 (0.525, 0.646)	**0.006**	0.181	2 days	**< 0.001**
ALB at admission	0.736 (0.683, 0.790)	**< 0.001**	0.384	36.35 g/l	**0.038**

Bold fonts represent statistical significance, *P* < 0.05; P* < 0.05 vs. PNI

*ALB* albumin, *AUC* area under the curve, *PNI* prognostic nutritional index

## Discussion

This study revealed that the PNI at admission, age, postoperative hospital stay, time to admission, hypertension, combined injuries and surgery type were independent factors for adverse perioperative outcomes in patients with femoral fractures, and the PNI at admission was likely a better independent predictor than the others. Our findings suggested that nutritional assessment at admission is necessary for patients with femoral fractures, and appropriate nutritional intervention should be considered for those patients.

Accumulating evidence indicates that approximately 20–40% of the patients show an acute, prolonged and profound decrease in postoperative serum ALB concentrations (Li and Chen 2019). This study showed that the mean ALB concentration in patients with or without adverse outcomes 1 day postoperatively were both < 30 g/l. This indicates that there may be a non-negligible nutrition risk in patients with femoral fractures during the perioperative period. However, these patients were administered parenteral nutrition interventions as routine care when the ALB concentration was < 30 g/l.

In this study, the multivariate regression analysis showed that the nutritional status at admission, not the postoperative nutritional status, was negatively correlated with adverse perioperative outcomes in patients with femoral fractures. Notably, the ROC curve analysis showed that the PNI at admission might provide better predictive value than other independent factors in this study, including age and time to admission. Though the ALB concentration is a primary measure included in the PNI (in addition to LYM count), the PNI at admission was superior to the ALB at admission in predicting adverse perioperative outcomes in patients with femoral fractures (*P* = 0.038). Malnutrition can be defined in various ways including by serological marker evaluation, anthropometric measurements and nutrition scoring tools. Amongst the various methods to define malnutrition, the most commonly used definition for malnutrition is an ALB level < 3.5 g/dL or a LYM count < 1500 cells (per mm^3^) (Morey et al. 2016). PNI, calculated by ALB and LYM, can represent the overall physiological functions and status of patients undergoing surgery, including nutrition, immunity and inflammation (Buzby et al. 1980; Li and Chen 2019; Onodera et al. 1984). Therefore, the PNI, a pre-treatment nutritional risk stratification tool, was better than ALB in predicting adverse perioperative outcomes. Additionally, it is well recognised that hypercoagulable state, stasis and endothelial injury contribute to the development of thrombosis. Patients with vascular endothelial injury caused by malnutrition may develop venous thrombosis through the aggregation of platelets or the release of cytokines from subendothelial tissue (Iguchi et al. 2020). Clinical evidence has shown that both hypoalbuminemia (Duman et al. 2019; Acharya et al. 2020) and lymphocytopenia (Kuplay et al. 2020; Yeung et al. 2021) are related to a high incidence of thrombus. Iguchi et al. found that preoperative PNI was a significant risk factor for the development of deep vein thrombosis after pancreatic surgery (Iguchi et al. 2020). In this study, we observed that thrombus accounted for 77.4% (123/159) of adverse perioperative outcomes. This finding suggested that PNI can be used to predict perioperative adverse outcomes in patients with femoral fractures. Based on the high correlation between nutrition and perioperative outcomes, this study suggested that patients with femoral fractures should undergo a nutritional assessment and nutritional intervention at admission, but not in the presence of malnutrition or hypoalbuminemia, or postoperatively.

Hypertension, which mostly occurs in the elderly, is related to vascular endothelial cell injury and is often accompanied by dyslipidaemia, and both vascular endothelial cell injury and dyslipidaemia are associated with the formation of venous thrombus. Thus, hypertension can be a risk factor for poor adverse outcomes in patients with femoral fractures. The type of surgery was classified into only two primary categories in this study: hemi/total-hip replacement and internal fixation (mainly consisting of intramedullary nailing, cannulated-screw and plate-screw internal fixation). The former is primarily performed in elderly patients with proximal femoral fractures, and the latter is performed commonly in younger or non-hip fracture patients. There are differences in incision, surgery duration, degree of ache, blood loss and hospital stay amongst patients treated by different surgical methods. In this study, we comprehensively evaluated the surgery-related factors, and the results indicated that the number of patients with femoral fractures who underwent internal fixation was 4.6 times the number of patients with femoral fractures who underwent hemi/total-hip replacement. The possible reasons were more severe pain, bleeding, inflammation, activity limitation and a longer bedridden period in the internal fixation-treated patients than in the hemi/total-hip replacement patients. However, postoperative hospital stay did not have a predictive value in this study because adverse postoperative outcomes lead to a prolonged postoperative hospital stay.

The NLR is considered a prognostic factor for outcomes and survival in cardiology, oncology and gastrointestinal surgery (Forget et al. 2015). It is also a risk factor for postoperative mortality and cardiovascular complications in elderly patients undergoing surgery for hip fracture repair (Forget et al. 2015 and Forget et al. 2016). The NEUT count, an effective and cheap inflammatory marker, is widely applied in clinical practice to guide diagnosis and therapy. In this report, we selected the NLR and NEUT count to represent perioperative inflammatory reaction. However, neither the NLR nor the NEUT count affected the adverse perioperative outcomes in patients with femoral fractures. This result may have been due to the administration of perioperative antibiotic prophylaxis in all patients, and because perioperative inflammation gets more attention than nutrition from surgeons and anaesthetists in China.

There are several limitations of this study. First, this was a single-centre study. Second, body mass index (BMI) was not evaluated in this study. BMI is an indicator for the assessment of nutritional status and a good predictor of morbidity and mortality (Miller et al. 2009); however, the height values were not documented in this study, mainly because patients with femoral fractures were unable to stand up to provide an accurate height measurement. Third, the lipid profile was not measured in most of the enrolled patients. Further studies are needed to evaluate the lipid profile (total cholesterol, triglycerides and lipoprotein concentrations) as the lipid profile is associated with the risk of venous thrombus (Garcia-Raso et al. 2013). Finally, we did not observe the long-term complications and mortality. Nevertheless, this study identified the risk factors and assessed the predictive value of PNI for adverse perioperative outcomes in patients with femoral fractures. Clinicians can use these easy-to-obtain and low-cost biomarkers to conduct risk assessments at admission or before an operation, which is helpful to early identify high-risk patients and give timely prevention and intervention.

## Conclusions

In conclusion, this study showed that age, hypertension, combined injuries and internal fixation were independent risk factors for adverse perioperative outcomes in patients with femoral fractures. Early admission to the hospital for treatment was associated with a decrease in the incidence of adverse perioperative outcomes. Most importantly, our findings suggested that all patients with femoral fractures require a nutritional assessment and appropriate nutritional intervention at admission, and that the PNI value at admission may be a good nutritional assessment indicator.

## Data Availability

The datasets analysed during the current study are available from the corresponding author on reasonable request.

## Abbreviations

PNI: Prognostic nutritional index
AUC: Area under the curve
ALB: Human serum albumin
ICD-10: International Classification of Diseases, tenth revision
ASA: American Society of Anesthesiologists
PAb: Prealbumin
GLB: Globulin
LYM: Lymphocyte
NEUT: Neutrophil
NLR: Neutrophil to lymphocyte ratio
OR: Odds ratio
CI: Confidence interval
BMI: Body mass index
